# Oxidative Phosphorus Chemistry Perturbed by Minerals

**DOI:** 10.3390/life12020198

**Published:** 2022-01-28

**Authors:** Arthur Omran, Josh Abbatiello, Tian Feng, Matthew A. Pasek

**Affiliations:** 1 Department of Chemistry, University of North Florida, 1 UNF Drive, Jacksonville, FL 32224, USA; 2 Department of Geosciences, University of South Florida, Tampa, FL 33620, USA; jabbatiello@usf.edu (J.A.); tianfeng1@usf.edu (T.F.); mpasek@usf.edu (M.A.P.)

**Keywords:** Fenton chemistry, reduced phosphorus, pyrophosphate, chemical complexity, chemical evolution, minerals, schreibersite, olivine, serpentinite, ulexite

## Abstract

Life is a complex, open chemical system that must be supported with energy inputs. If one fathoms how simple early life must have been, the complexity of modern-day life is staggering by comparison. A minimally complex system that could plausibly provide pyrophosphates for early life could be the oxidation of reduced phosphorus sources such as hypophosphite and phosphite. Like all plausible prebiotic chemistries, this system would have been altered by minerals and rocks in close contact with the evolving solutions. This study addresses the different types of perturbations that minerals might have on this chemical system. This study finds that minerals may inhibit the total production of oxidized phosphorus from reduced phosphorus species, they may facilitate the production of phosphate, or they may facilitate the production of pyrophosphate. This study concludes with the idea that mineral perturbations from the environment increase the chemical complexity of this system.

## 1. Introduction

Life is the ultimate example of a complex chemical system [1,2]. As such, it must be buttressed by energetic inputs to maintain homeostasis. Life requires energy, and present-day life uses phosphates such as adenosine triphosphate (ATP) or acetyl-phosphate as carrier molecules to transfer such energy [2]. In a completely simplistic system that one may fathom to “start from scratch,” organophosphates such as nucleotide triphosphates are too sophisticated or evolved [3] and are likely products of an RNA world and the work of earlier metabolism [3]. A complex system may evolve organophosphates but perhaps not as the initial energy currency. Various inorganic phosphate species may have contributed to the prebiotic formation of protometabolic systems [4,5], at least if phosphorus is critical to life’s origin [6]. Few simple phosphorus species exist in nature that may have supported life early on, such as reduced phosphorus species, phosphates (PO_4_^3−^), and polyphosphates [4].

**Scheme 1:**
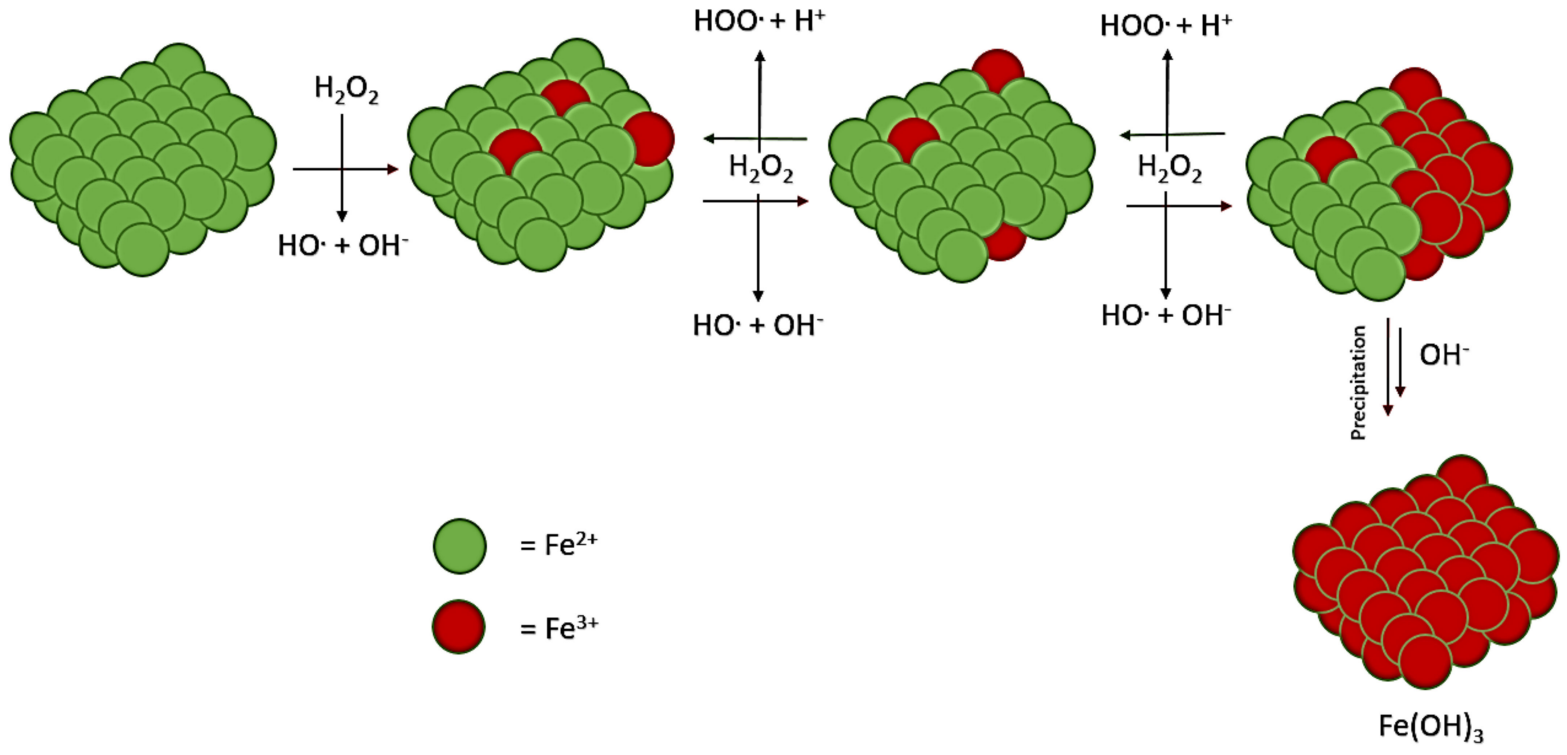
Explanation of the Fenton reaction. Reaction of the iron complex is partially soluble and in solution, with iron (III) likely being solid and iron (II) more soluble.

A far-from-equilibrium system with environmental energy input could enable endergonic reactions. Life would need a system that readily liberates solvated electrons and provides a soluble phosphorus source from the environment. One such system that could provide electrons for redox chemistry may be the Fenton system. In this system, iron (II) ions in solution, or in a scaffolding, break down aqueous hydrogen peroxide into hydroxyl radicals (Scheme 1) [7,8,9,10,11,12]. Throughout this work, we represent this reaction using schemes of the imagined scaffolds in solution; this does not preclude simple, individual soluble ions of iron (II) from taking part in the reaction.

When coupled to the oxidation of reduced phosphorus species, such as hypophosphite (H_2_PO_2_^−^) and phosphite (HPO_3_^2−^), these hydroxyl radicals, liberated from the Fenton reaction, oxidized these reduced P compounds to phosphate and pyrophosphate (P_2_O_7_^4−^) [13,14,15]. The reduced phosphorus species themselves are soluble compared to phosphate, which precipitates readily in the presence of divalent and trivalent metal cations [4,5,13,14]. Pyrophosphate may be evolutionarily or prebiotically important due to its being a potential precursor to ATP in the evolution of life [16,17,18,19,20]. Therefore, pyrophosphate may have been life’s original energy currency, though this is debated [16,17,18].

**Figure 1:**
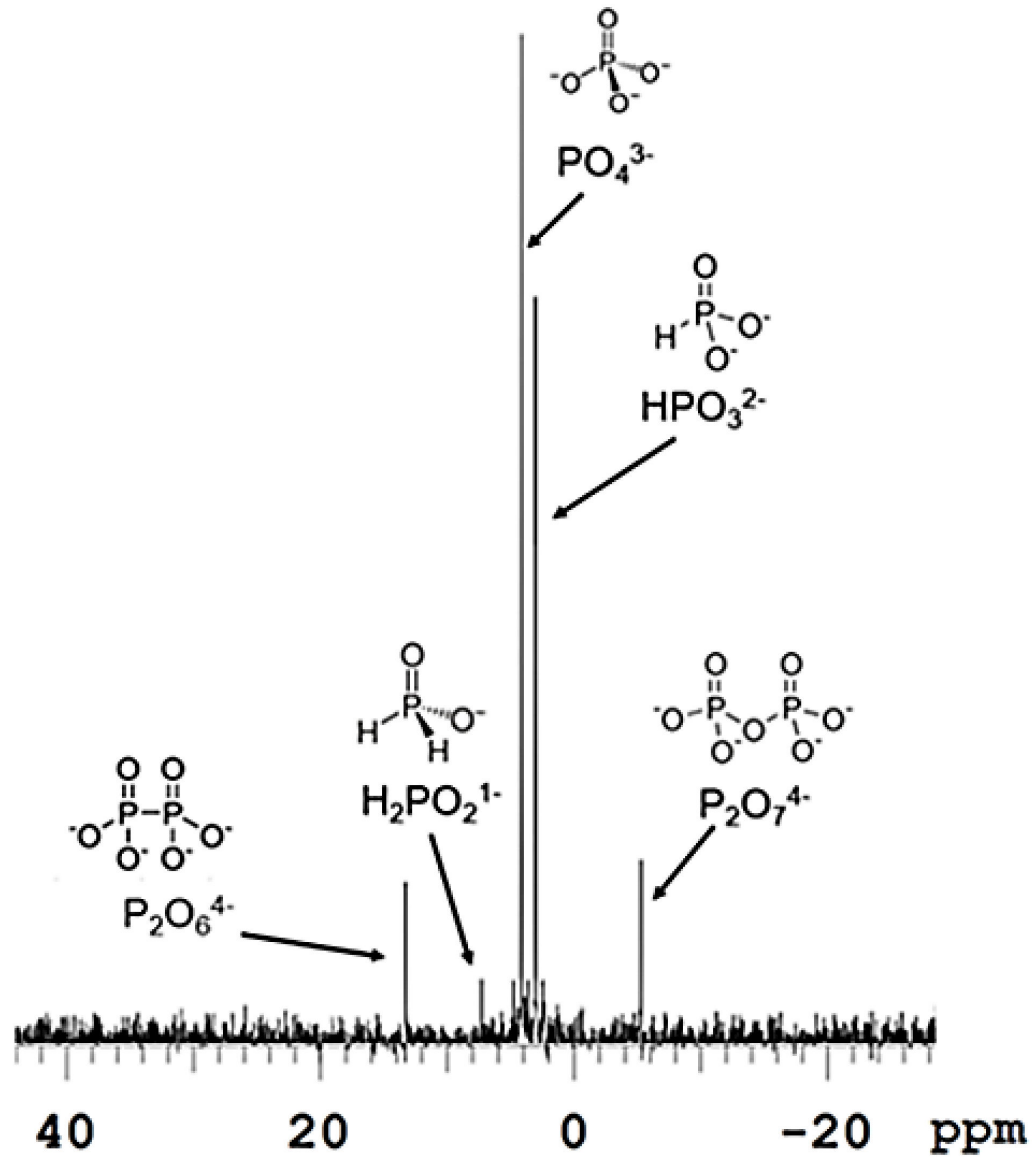
P^31^-NMR spectrum of the corrosion of schreibersite. An amount of 0.5 Fe_3_P powder was placed in 15 mL of water for 1 week under a 20% mixed CO_2_/CH_4_ and 80% N_2_ atmosphere. The water contained multiple phosphorus species, including reduced phosphorus species, such as hypophosphite (H_2_PO_2_^−^) and phosphite (HPO_3_^2−^).

This Fenton system, coupled with reduced phosphorus, may be a plausible system on rocky planets, such as the early Earth [5,14,15]. Firstly, the reduced P may have been provided by the meteoric input of iron meteorites, which are rich in the mineral schreibersite ((Fe/Ni)_3_P), or from lightning strikes which may have formed schreibersite as well [13,21]. This mineral is a highly reduced P source that, when exposed to water, corrodes and forms reduced P species such as hypophosphite (H_2_PO_2_^−^) and phosphite (HPO_3_^2−^), among other phosphate species (Figure 1) [13].

Secondly, hydrogen peroxide may have been plentiful on early rocky planets as one of the earliest oxidants. It may have formed via the photolysis of atmospheric water and rained down on the surface [22,23,24]. Additionally, peroxides form on the surface of icy moons, such as Europa or Enceladus, due to solar radiation of surface ice on the moons [25,26,27]. It is with these P, iron, and peroxide resources in mind that one may view the Fenton chemical system as plausible towards the emergence and chemical evolution of life.

A complex system is a system comprising many components with much interconnectedness and many relations between the system’s components. This system has multiple components, iron species, P species, peroxides, etc., and the reactions affect the various species as well. As a complex system, this P-coupled Fenton chemistry is vulnerable to “dirty” chemistry, as are all plausible prebiotic chemistries. We therefore wish to understand the influence of minerals in the environment on this system.

## 2. Materials and Methods

Solutions of Fenton’s reagent, 10 mL, were aliquoted into 20 mL borosilicate vials. Fenton’s reagent was synthesized using 100 mM of FeCl_2_ solution (Fischer, Waltham, MA, USA). The solutions contained a final concentration of 880 mM of H_2_O_2_ (Fischer); the peroxide was added very slowly. The solution also contained 100 mM of Na_2_HPO_3_ or 100 mM of NaH_2_PO_2_ (both from Sigma-Aldrich, St. Louis, MO, USA) as an initial phosphorus source. The H_2_O_2_ was added after the FeCl_2_ and the P species.

In addition to the components of a Fenton solution and a phosphorus source, we added minerals individually to see if they influenced the product distributions. Each experiment contained a 0.05 g sample of a mineral or rock, including diopside, magnetite, serpentinite, quartz, kaolinite, siderite, newberyite, hematite, ulexite, schreibersite, orthoclase, olivine, hydroxyapatite, and struvite (Table S1). We selected these minerals based on the minerals’ likely presence during the Hadean Eon or their prebiotic plausibility/utility (Table S1). The final experiment had no minerals as a control. Reactions took place at room temperature (25 °C) and were gently stirred for 48 h. Solutions were slowly titrated over a period of 5–10 min with 1 mL of 12 M NaOH. Fenton solutions were then filtered through 0.2 µm pore nylon syringe filters (Thermo Scientific, Waltham, MA, USA) using 10 mL Hamilton luer lock syringes.

An amount of 500 µL of each solution was aliquoted into NMR tubes, along with 10% by volume D_2_O (Sigma-Aldrich, St. Louis, MO, USA) used to lock spectral measurements. Solutions were characterized using ^31^P NMR on an INOVA Unity 400 MHz NMR. The INOVA 400 was run at 161.84 MHz, with 2052 normal scans per sample in H-coupled-NOE mode with a 1 s delay between acquisitions. The spectral width was 200 ppm, and the running temperature was set at 25 °C. The limit of detection for phosphorus species in solution on the INOVA 400 was 50–80 µg/g, well below our measurements and concentrations. All data analyses were run on SpinWorks 4.0 software and SigmaPlot 10.0 software.

Samples of the solution phases were also analyzed via inductively coupled plasma–optical emission spectrometry (ICP–OES) before undergoing a mineral extraction. Filtrates of the minerals in solution and the precipitated iron hydroxides were extracted using a 4:1 NaOH EDTA extraction solution. Filtrates were extracted for one week while stirring at room temperature, as described above. Extracts were collected and inspected via NMR, as stated above, and analyzed via ICP–OES.

Solution phase or mineral phase was analyzed via ICP–OES. The analysis was performed on the Avio 200 ICP–OES from PerkinElmer with a 1.5 mm loop. The internal standard was 100 ppm germanium. The calibration standards used were 0.02, 0.1, 1, 5, 50, and 100 ppm of phosphoric acid mixed with deionized water. A 10 ppm phosphorus acid standard, as well as a blank of deionized water, were used as quality controls throughout the run. The samples were all diluted 100-fold, and then run on the ICP–OES to measure P content.

## 3. Results and Discussion

**Scheme 2:**
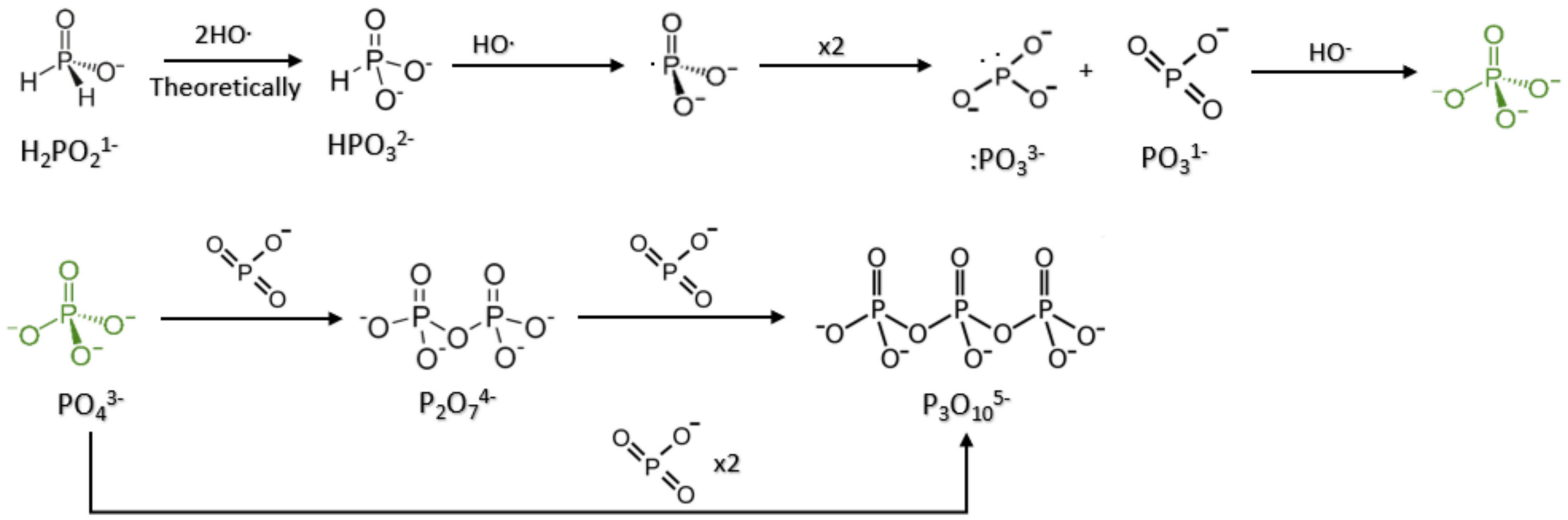
Explanation of the phosphorus oxidation pathway.

**Figure 2:**
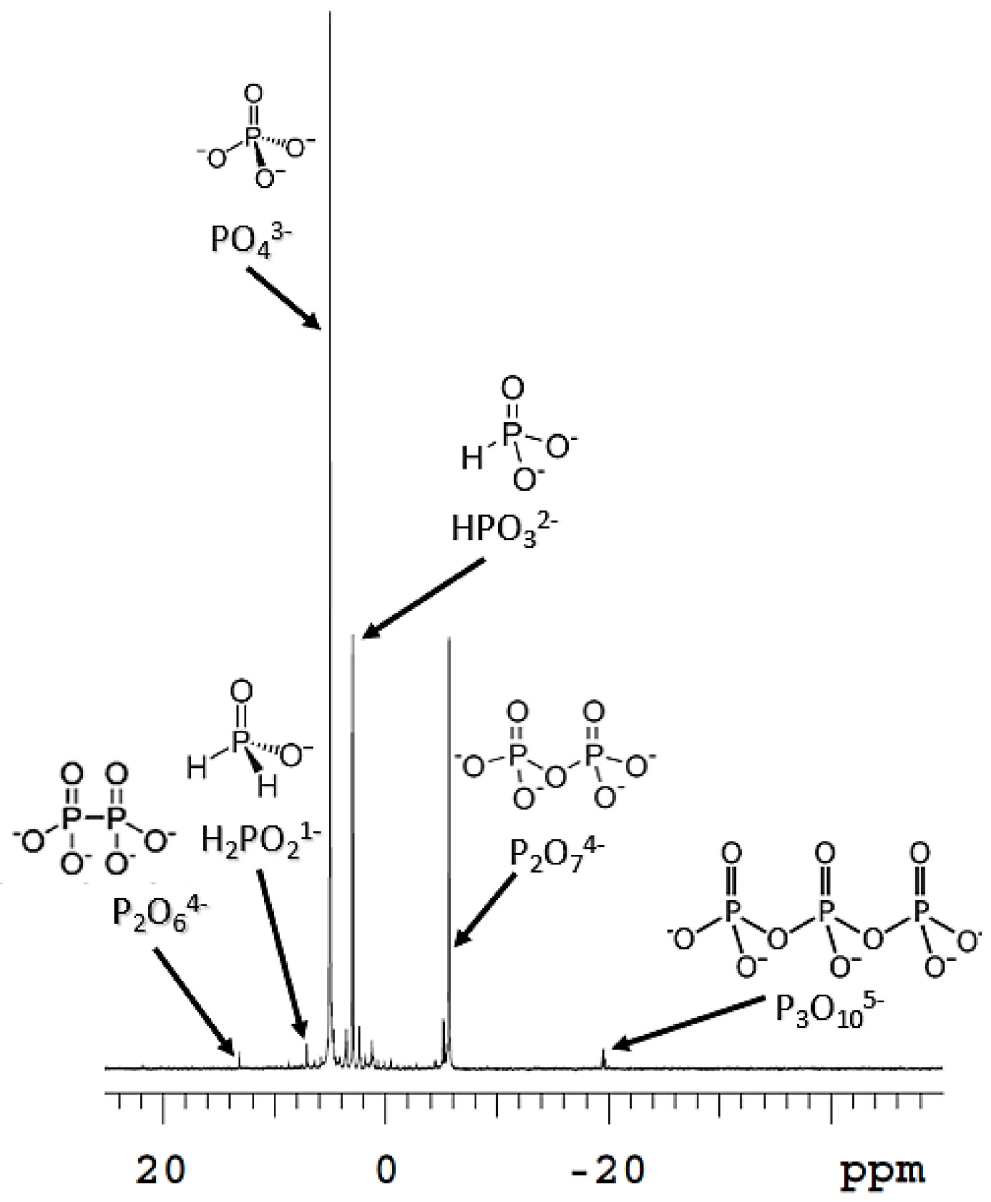
Complex mixture of different oxidation states of P species. Hypophosphite added to the Fenton reaction in the presence of schreibersite. Decoupled ^31^P NMR.

The Fenton system generates radicals from peroxide. These hydroxyl radicals react with P-H bonds to generate P oxidation and polyphosphates (Scheme 2, Figure 2). Depending on which reduced P source is initially placed in the environment, this system will generate a complex mixture of P species at different redox states (Figure 2). If one starts with hypophosphite in solution, the products include phosphite, phosphate, pyrophosphate, and small amounts of hypophosphate, triphosphate, and pyrophosphite (rarely) (Figure S1, Table S2). Phosphite as a P source in Fenton reactions produced phosphates and pyrophosphate (with some triphosphate in small amounts) (Table 1, Table 2 and Table 3).

The Fenton chemistry requires soluble iron in solution to decompose hydrogen peroxide; it, too, can function as part of a heterogenous catalyst acting as a peroxide-decomposing scaffold in solution (Scheme 1) [7,8,9,10,11,12]. The preliminary work was carried out with hypophosphite solutional systems; increased oxidation of phosphorus species in solution was observed in the presence of hydroxyapatite and schreibersite (Table S2). After those experiments, this study mainly focused on phosphite undergoing oxidation due to the Fenton system. We verified that the use of insoluble iron mineral species without any soluble iron results in no reaction by adding powdered magnetite with no added soluble iron (FeCl_2_) in solution, and we observed no to little (<5%) P oxidation to phosphate under such conditions.

A criticism of the Fenton system is that it may be harmful to organic compounds and degrade them. Indeed, Fenton’s reagent was developed by Henry Fenton in 1894 as an analytical agent to detect organics such as tartaric acid [7]. It is currently used as a green chemical solution to clean up and break down organics [10,11]. In these experiments, no organics were added as substrates; thus, no degradation occurred, but phosphorylation of organic substrates would likely require addition of the organic after the Fenton reaction has ceased. For example, surface pooling or small ponds that receive peroxide downpour could plausibly house these reactions, producing phosphates and pyrophosphates. Any organics introduced into these ponds after the reactions completed might then be phosphorylated due to other environmental factors, such as UV light.

### 3.1. Mineral Perturbation: Inhibition

The phosphite-added Fenton reactions had diverse outcomes. Firstly, the control group that had 100 mM of NaHPO_3_ and 100 mM of FeCl_2_ produced 53% phosphate, 23% pyrophosphate, and ~3% triphosphates, with 21% phosphite left. The no-mineral-added control was used as a benchmark to compare against the mineral-added reactions. Magnetite, serpentinite, and orthoclase seemed to inhibit the oxidation of the phosphite in the Fenton solution phase (Table 1). The least amount of P oxidation occurred with these minerals present, with about 30–40% of the phosphite left unreacted in solution.

**Figure 3:**
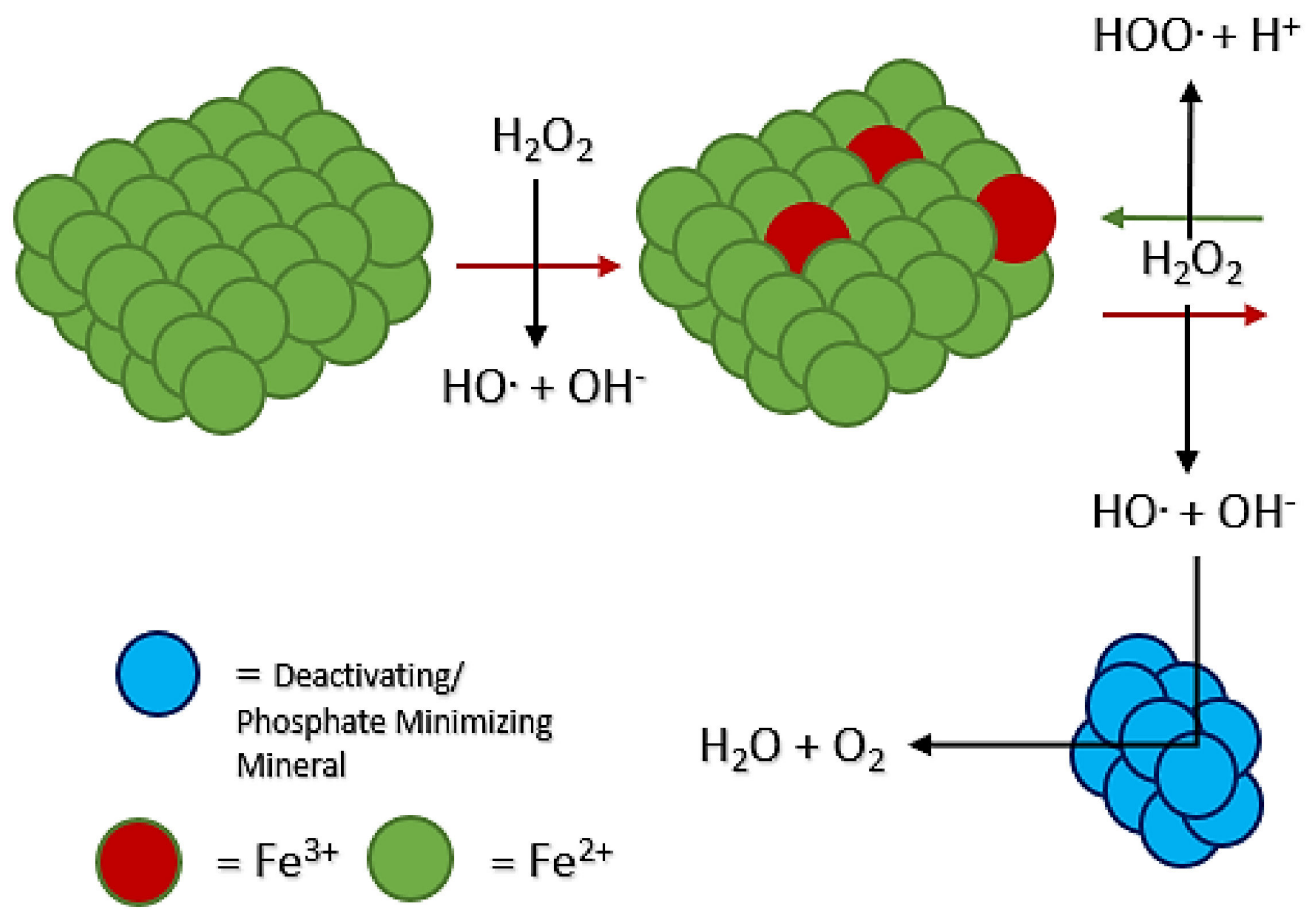
Diagram of how minerals may inhibit the Fenton reaction.

It is possible that an antioxidant mechanism, such as scavenging the peroxide radicals, is involved in this result. As the Fenton system (Scheme 1) occurs and radicals are generated, a mineral in the environment may act to inhibit the system by using up the radicals before they are used to fuel P oxidation (Figure 3). Both serpentinite and magnetite are Fe^2+^-containing rocks and minerals. Likely, stable products would include water and oxygen gas (Figure 3).

### 3.2. Mineral Perturbation: Phosphate Promotion

**Figure 4:**
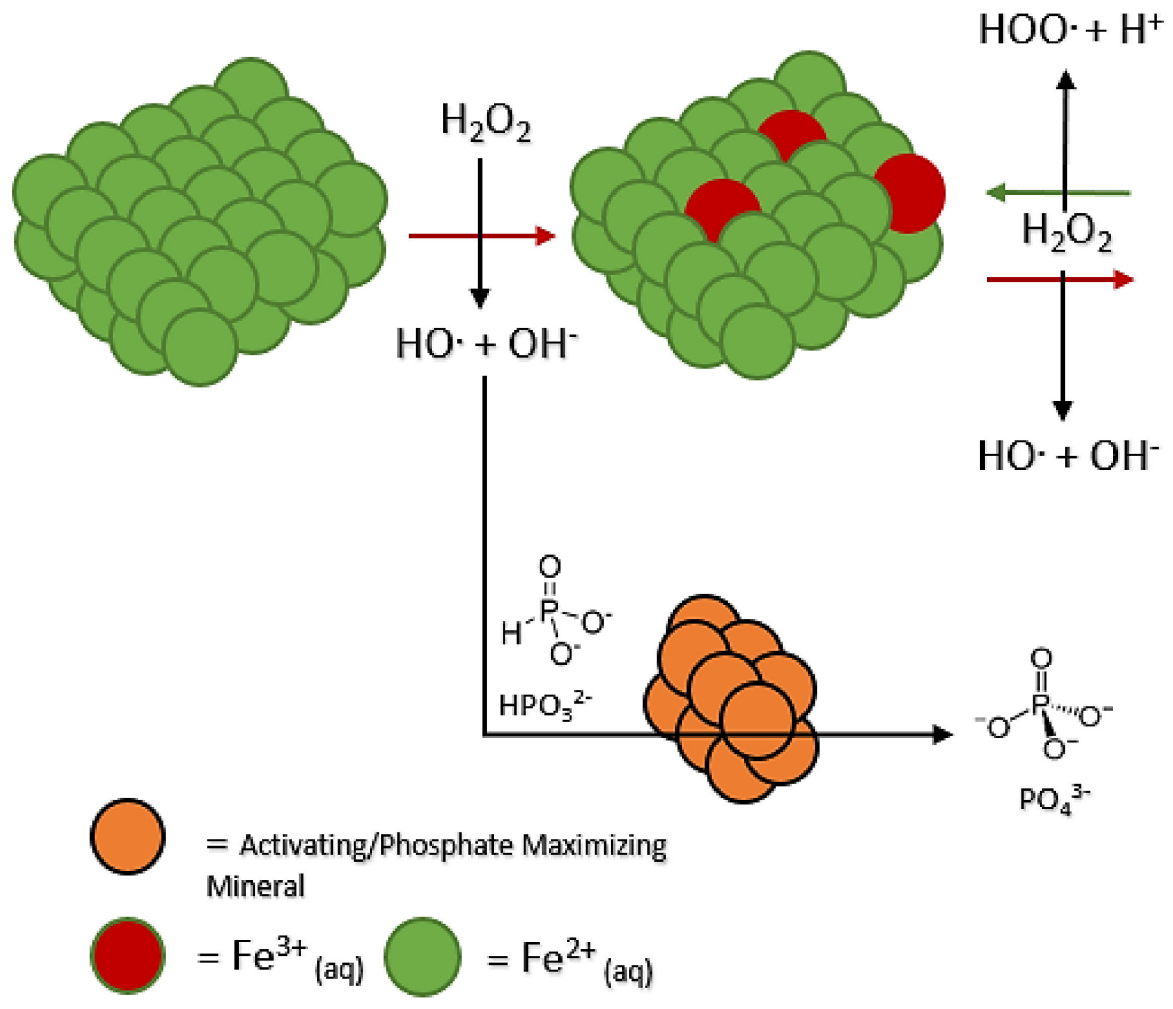
Diagram of how minerals may enhance the Fenton reaction.

Another outcome observed was the generation of more phosphate in solution than phosphite (Table 2). Some minerals managed to perturb the system towards 60–78% phosphate, but not a great amount more; these included newberyite, calcite, diopside, gypsum, hydroxyapatite, and ulexite (Table 2). The gypsum mineral-added systems did not exhibit an increase in total oxidation, with unreacted phosphite present at a concentration like that of the control. One must then assume this phenomenon or mineral “influence” to be based on the minerals interacting with the phosphorus species, and not just the peroxide in solution (Figure 4). Interestingly, some minerals showed a similar influence as well as increased total oxidation of phosphite (Table 2); these minerals included calcite, newberyite, hydroxyapatite, diopside, and ulexite, with 90+% total oxidation of phosphite in solution. One may suspect better phosphite reactivity towards hydroxyl radicals in addition to the mineral working on the phosphite itself (Figure S2).

### 3.3. Mineral Perturbation: Pyrophosphate Promotion

**Figure 5:**
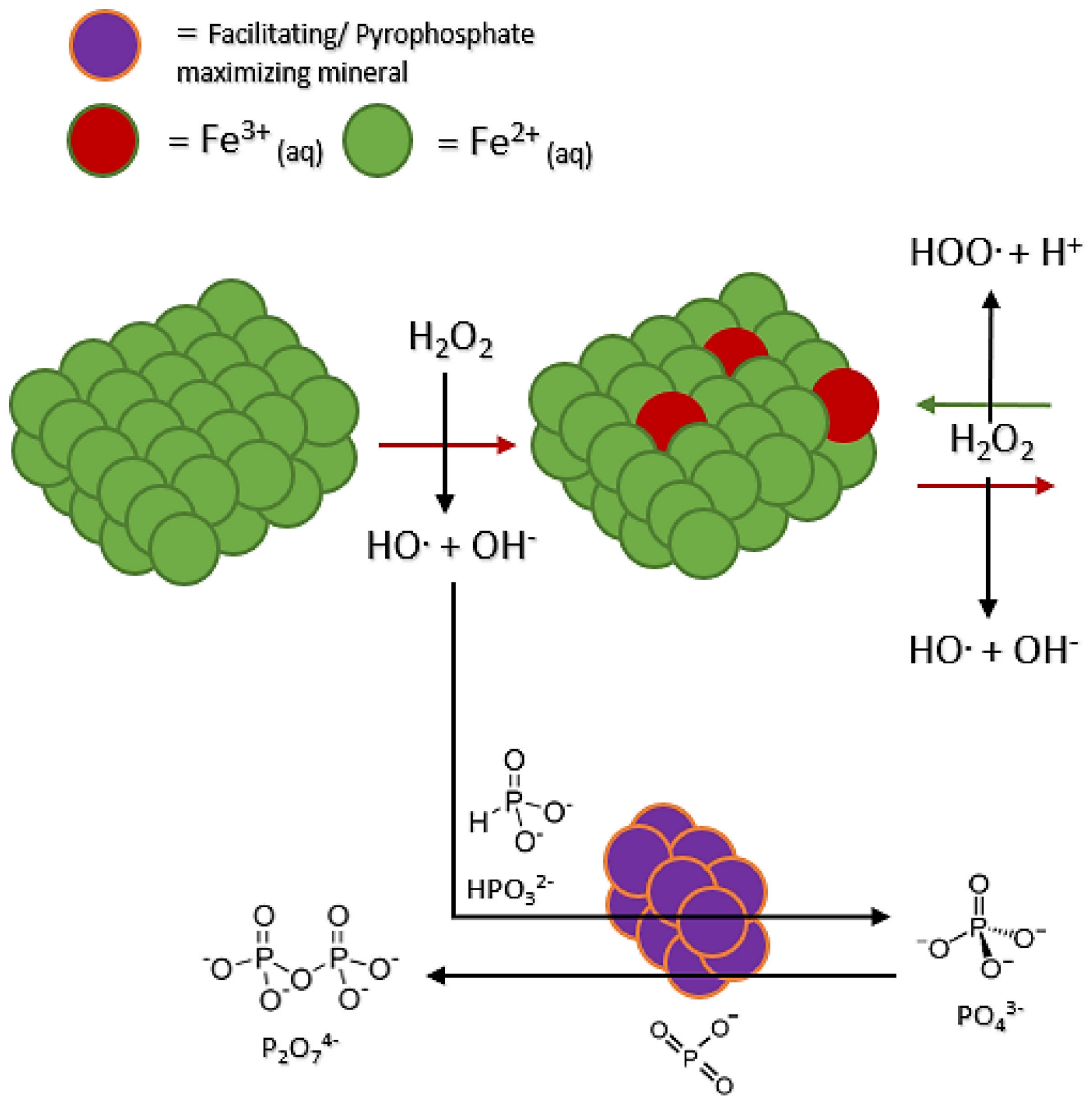
Diagram of how minerals may be facilitating phosphorus chemistry via the Fenton reaction.

A third type of influence or perturbation was observed. Like the phosphate increase observed, some minerals appear to increase the amount of pyrophosphate (Table 3). Diopside, kaolinite, sand (quartz), and hematite showed slight increases of pyrophosphate in solution. Schreibersite, siderite, and struvite showed the greatest production of pyrophosphate at 30+%, with marked increases in total oxidation compared to the control. We observed the increased total oxidation of phosphorus for each of these mineral groups. Diopside, kaolinite, sand (quartz), and hematite all showed 27+% production of pyrophosphate in solution. It could be that these minerals directly bind the phosphorus in solution. As the Fenton system proceeds, the phosphorus species cluster together on the mineral surface, facilitating the production of pyrophosphate and other polyphosphates (Figure 5). Additionally, accompanying increases in total oxidation are slight increases in detected triphosphate species (Table 3). Overall, triphosphate does not seem to be affected like phosphate or pyrophosphate, potentially due to its breaking down into smaller P species or being at a low enough concentration that variations are hard to detect.

The phosphorus studied in this system is specifically the phosphorus in aqueous solution that can be detected via NMR. The phosphorus adsorbed to the mineral phase was also inspected. Schreibersite had triphosphate and hypophosphate in the mineral phase, differing from the other minerals. This is likely due to the corrosion of the schreibersite itself while in aqueous solution, which is known to produce these anions [28]. Struvite had a noted increase in pyrophosphate. Struvite, being a phosphate-bearing mineral, likely added phosphate to the reactions. Struvite’s mineral phase adsorbed about 50+% additional pyrophosphate, likely due to the reaction of phosphates with phosphite in the mineral phase.

### 3.4. Additional Verifications

When compared to standards of our phosphorus reagents or to no reaction, we saw no oxidation of phosphorus or oligomers of phosphorus, similarly to what we saw in the absence of soluble iron. It is important to mention as well that olivine did not seem to influence the product distributions, so some minerals do not influence the reactions (Table S4). In terms of reproducibility, these reactions are chaotic in that they rely on free radical formation from peroxide breakdown. These reactions in general can be compared to one another within datasets as long as the same peroxide solution was used at the same concentrations at roughly the same time. With that in mind, we can clearly see within the datasets the influence of our minerals on the Fenton system. A plethora of diverse oxidized P species was observed; this included pyrophosphite on rare occasions (Figure S1, Table S2).

Obviously, the chemical composition of the minerals matters in their effect on the system, in this study we focused on the product distributions mostly, looking for overarching trends on the system holistically. We did note additional trends based on mineral composition. Minerals have various compositions and absorptivity (Tables S3 and S4). The adsorptions of P in the mineral phase vs. the solution phase was inspected via ICP–OES (Table S3). Mineral stability and reactivity matter in terms of P recovery in solution and in the mineral phase itself. Very stable samples, such as our serpentinites, were shown to be less perturbing than minerals with greater solubility or reactivity, such as hydroxyapatite. We expected to see such variation based on the diversity of mineral and rock species selected (Table S1).

The majority of our starting phosphorus was accounted for in the solution and mineral phase using ICP–OES, ranging from ~70% to 120%. Samples with lower P detection were still in acceptable ranges for ICP–OES, allowing us to track where the P in the system was mostly located. The lower detections may be due to incomplete extraction with the mineral phase, so some P would cling to the mineral and not be accounted for. Additionally, some of the P might get lost in the iron oxides formed by the Fenton process. Most of the P was present in the original filtrate, as the filtrate had P contents of 51–117% of the initial added P (with the exception of hydroxyapatite, which bore sparingly little P). The extracted P in contrast was usually 1–18% of the total added P. Schreibersite, struvite, and newberyite had a higher total phosphorus content via ICP–OES due to the release of P from these minerals, accounting for the 100+% P detected (Table 3). In addition, the extracts tended to have a higher abundance of pyrophosphate vs. phosphate, suggesting that pyrophosphate binds to the mineral surface. In contrast, the phosphite to phosphate ratios are generally similar in both the filtrates and the extracts, although we note that, in general, this ratio is more varied in those samples that released more P upon extraction (e.g., hydroxyapatite). We note as well that serpentinite had more phosphite than phosphate in both the solution phase and the extraction, suggesting either a phosphite source in the serpentinite, or an impedance of phosphite oxidation via the serpentinite. It is possible the serpentine rock exchanges Mg for Fe during the Fenton process, especially if the rock is rich in brucites. Further study is needed for serpentinite regarding its relationships with reduced P species.

## 4. Conclusions

Phosphorus species pertain to a great many minerals, of various physical properties and reactivities, valuable to prebiotic chemistries [4,5,28,29,30,31,32]. Not only has reduced phosphorus been detected in meteorites, but also in rocks and minerals originating here on Earth. Other complex systems seem viable in multiple environments, [33,34,35] with some models incorporating phosphorus into self-assembling systems [36].

The Fenton system induces an oxidation of reduced P to phosphate and polyphosphates and is a complex chemical system. This chemical system is a plausible prebiotic system based on the simplicity and plausibility of its reagents. This chemical system may increase in complexity based on the minerals in its “dirty” environment. Those minerals are additional components, after all, contributing to systemic complexity. Complexity was measured by the increase in the distribution of types of P species in solution. These P species increased in their oxidation states and increased in their molecular weights as compared to the reduced P species we started with. There is a limit to the system’s complexity in that complex organics are vulnerable to Fenton chemistry. Tolerance to peroxides may have been necessary for life originally [24].

Many types of perturbations were observed. The perturbations are based on the diversity of mineral species and compositions. These include promoting the formation of phosphate, promoting the formation of pyrophosphate, or stymieing the oxidative process altogether. The importance of the “dirty” environment and the “messy” product distributions may be fundamental in our understanding prebiotic chemistry in terms of chemical complexity. The minerals in the environment increase the chemical space of possibility for this open system and may even act as switches or controls for an open system and its environment. In a multimineral system or microenvironment, these switches may add together or oppose each other, totaling to a sum perturbation for the system, determining the systems’ product distribution. Future work with multiple minerals per reaction system is needed to explore this.

## Figures and Tables

**Table 1:** Mineral-added Fenton reactions with phosphite. Minerals inhibiting the Fenton reaction. All numeric values are integral percentages taken from NMR of single-batch simultaneous reactions.

Mineral	Phosphite	Phosphate	Pyrophosphate	Triphosphate
None	21.6	52.6	23.2	2.6
Magnetite	31.6	40.9	25	2.5
Orthoclase	39.7	47.8	11.5	1
Serpentinite	73.5	18.1	8.2	0.2

**Table 2:** Mineral-added Fenton reactions with phosphite. Minerals enhancing the Fenton reaction. All numeric values are integral percentages taken from NMR of single-batch simultaneous reactions.

Mineral	Phosphite	Phosphate	Pyrophosphate	Triphosphate
None	21.6	52.6	23.2	2.6
Calcite	4.5	75	19.7	
Diopside	5.2	64	27.3	2.6
Gypsum	21.7	63.7	14	0.6
Hydroxyapatite	3.8	77	18.5	0.6
Newberyite	3.1	78.7	18.2	
Ulexite	1.5	73.5	25	

**Table 3:** Mineral-added Fenton reactions with phosphite. Minerals facilitating phosphorus chemistry via the Fenton reaction. All numeric values are integral percentages taken from NMR of single-batch simultaneous reactions.

Mineral	Phosphite	Phosphate	Pyrophosphate	Triphosphate
Control (None)	21.6	52.6	23.2	2.6
Hematite	19.7	49.3	28	3
Kaolinite	15	53.5	28.3	3.2
Sand	14.2	54.7	28.4	2.7
Schreibersite	11.5	52	33.3	3.1
Siderite	17.6	48.7	30.7	3
Struvite	7.6	47.4	44.1	0.9

## Data Availability

Not applicable.

## Supplementary Materials

The following supporting information can be downloaded at: https://www.mdpi.com/article/10.3390/life12020198/s1. Figure S1: Fenton reaction with serpentinite and hypophosphite added, Figure S2: Additional possible Fenton reaction diagram, Table S1: List of plausibly prebiotic minerals, Table S2: Hypophosphite added Fenton reactions solutional product distribution table, Table S3: ICPOES data, Table S4: Mineral extraction NMR data.
