# Supplementary material for: Oxidative Phosphorus Chemistry Perturbed by Minerals
Source: Life (Basel). 2022 Jan 28;12(2):198. doi: 10.3390/life12020198 (PMC8878404; doi:10.3390/life12020198)
Supplement: Supplementary file 1 [file life-12-00198-s001.zip › life-1553777-supplementary.pdf]

Supplementary

Oxidative Phosphorus Chemistry Perturbed by Minerals

By Arthur Omran [1,2,*], Josh Abbatiello [2], Tian Feng [2] and Matthew A. Pasek [2]

[1] Department of Chemistry, University of North Florida, 1 UNF Drive, Jacksonville, FL 32224, USA
[2] Department of Geosciences, University of South Florida, Tampa, FL 33620, USA; jabbatiello@usf.edu (J.A.); tianfeng1@usf.edu (T.F.); mpasek@usf.edu (M.A.P.)
* Correspondence: N00431947@unf.edu

Table S1. List of Plausibly Prebiotic Minerals.

Name	Group	IMA Formula	Occurrence*	Reference
Diopside	Silicate	$CaMgSi_2O_6$	CM,MA,MT,RM,UM	[1]
Kaolinite	Silicate	$Al_2Si_2O_5(OH)_4$	HY	
Orthoclase	Silicate	$KAlSi_3O_8$	GR,MA,SD,UM,ZR	
Antigorite	Silicate	$Mg_3Si_2O_5(OH)_4$	CM,HY,SP,UM	
Forsterite	Silicate	Mg_2SiO_4	CM,MA,MT,UM,UR	
Quartz	Oxide	SiO_2	GR,HY,MT,SD,ZR	
Hematite	Oxide	Fe_2O_3	CM,GR,HY,RM,VP	
Magnetite	Oxide	Fe_3O_4	CM, GR, MA, MT, RM, SD, UM	
Schreibersite	Phosphide	$(Fe,Ni)_3P$	MT	
Gypsum	Sulfate	$CaSO_4{\cdot}2H_2O$	EV, LT, MT, PR, VP	
Siderite	Carbonate	$FeCO_3$	AK, AU, GR, HY, UM	
Calcite	Carbonate	$CaCO_3$	AK,HY,MA,MT,PR	
Hydroxyapatite	Phosphate	$Ca_5(PO_4)_3OH$	HY,SP	
Newberyite	Phosphate	$MgHPO_4{\cdot}3H_2O$	HY	[2]
Struvite	Phosphate	$MgNH_4PO_4{\cdot}6H_2O$	HY	
Ulexite	Borate	$NaCaB_5O_6(OH)_6{\cdot}5H_2O$	N/A	[3]

* Modes of Occurrence: AK: alkali igneous; AU: authigenic; CM: contact metamorphism; EV: evaporite; GR: granitoid igneous; HY: hydrothermal alteration; LT: low-temperature metamorphism; MA: mafic igneous; MT: meteorite; PR: aqueous precipitate; RM: regional metamorphism; SD: clastic sedimentary environment; SP: serpentinization; UM: ultramafic igneous; UR: "ur-minerals" from pre-solar grains; VP: vapor phase deposition at volcanic fumaroles and other pneumatolytic processes; ZR: inclusions in Hadean zircons.[1].

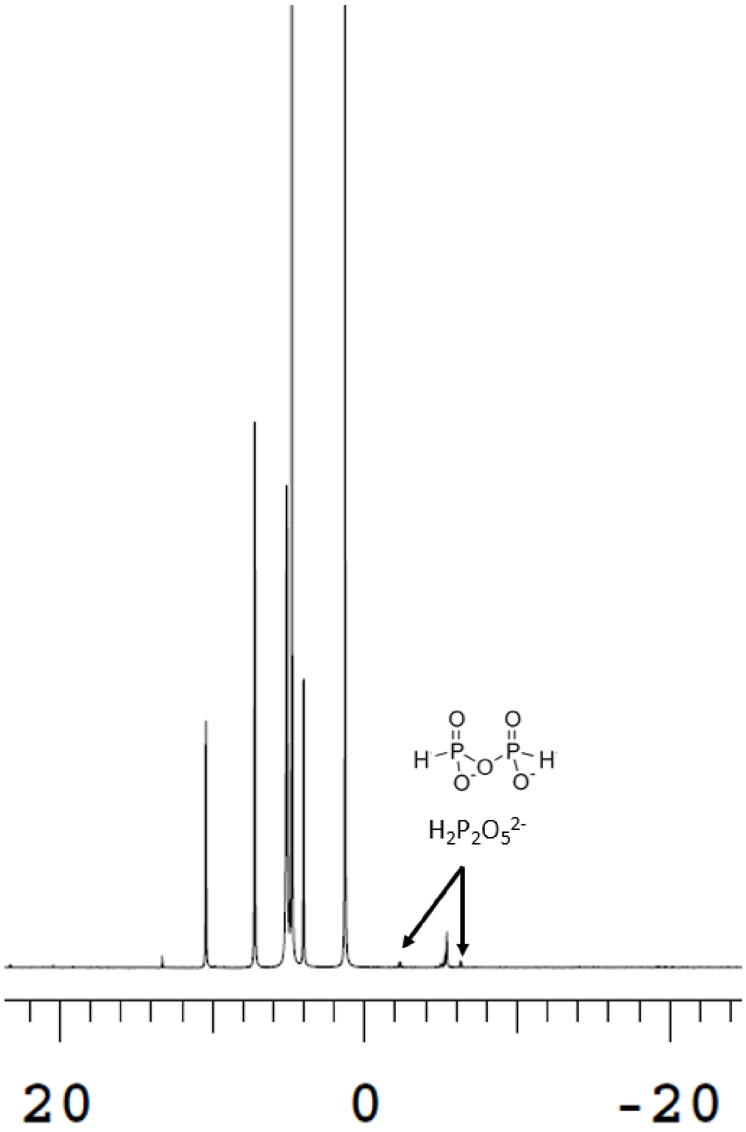

Figure S1. Fenton reaction with serpentinite and hypophosphite added. Peaks for pyrophosphite detected at -2.2 and -6.2 ppm.

Table S2. Hypophosphite Added Fenton reactions solutional product distribution table. All measurements are from peak integrations in the P31-NMR spectra.

No Minerals		Int. %	Serpentinite		Int. %	Schreibersite		Int. %
**1**	**$H_2PO_2^{1-}$**	**23.1**	1	$H_2PO_2^{1-}$	23.6	1	$H_2PO_2^{1-}$	0.5
2	HPO_3^{2-}	47.1	2	HPO_3^{2-}	50.7	2	HPO_3^{2-}	20
3	PO_4^{3-}	28.5	3	PO_4^{3-}	23.1	3	PO_4^{3-}	57.6
4	$P_2O_7^{4-}$	1.1	4	$P_2O_7^{4-}$	1.1	4	$P_2O_7^{4-}$	21.2
5	$P_2O_6^{4-}$	0.2	5	$P_2O_6^{4-}$	0.03	5	$P_3O_{10}^{5-}$	0.5
			6	$H_2P_2O_5^{2-}$	0.34	6	$P_2O_6^{4-}$	0.2
Hydroxyapatite		Int. %	Kaolinite		Int. %	Sand		Int. %
1	$H_2PO_2^{1-}$	11.5	1	$H_2PO_2^{1-}$	20.6	1	$H_2PO_2^{1-}$	22.6
2	HPO_3^{2-}	41	2	HPO_3^{2-}	48.1	2	HPO_3^{2-}	49.4
3	PO_4^{3-}	43.7	3	PO_4^{3-}	29.8	3	PO_4^{3-}	26.4
4	$P_2O_7^{4-}$	3.7	4	$P_2O_7^{4-}$	1.2	4	$P_2O_7^{4-}$	1.3
5	$P_2O_6^{4-}$	0.03	5	$P_2O_6^{4-}$	0.2	5	$P_2O_6^{4-}$	0.3

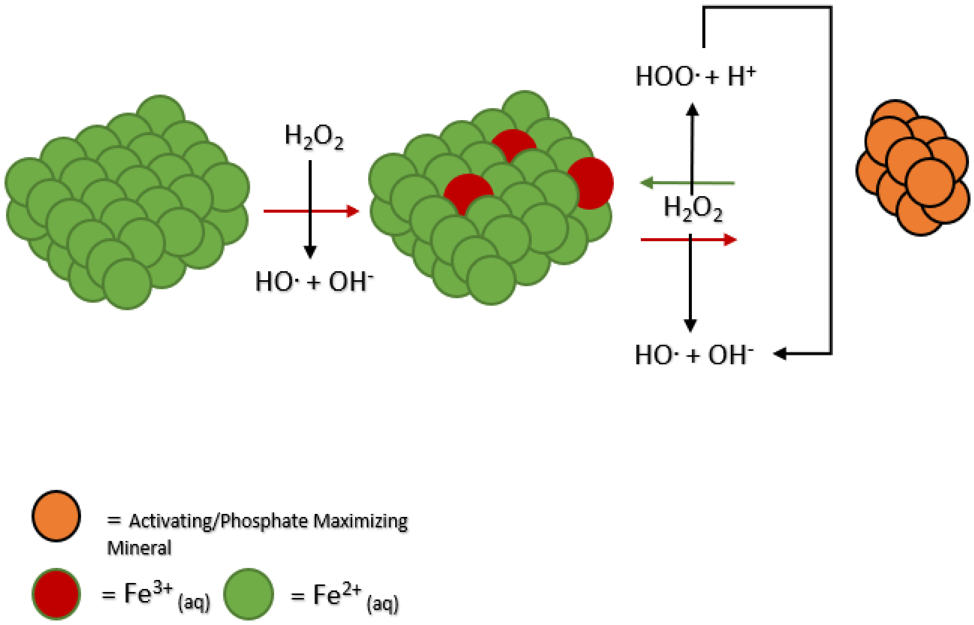

Figure S2. Additional Possible Fenton Reaction Diagram. Fenton reaction diagram showing a possible mechanism for increased oxidation by phosphate increasing minerals.

Table S3. ICPOES Data. The numbers refer to the counts for the solution phase P (before EDTA/NaOH extraction), mineral phase extracts (after EDTA/NaOH extraction), and extract/solution (count 1 divided by count 2). This is meant to show a before and after treatment so we can get an idea of the amount of P in solution and the amount of P in the mineral.

ICP OES Data					
	Solution Phase	Mineral Phase Extracts	Total P molarity	Fraction of P recovered (%)	Extract/Solution
Blank	9.17386E-06	2.14883E-05	3.06622E-05		
Calcite	0.051133892	0.011530277	0.062664169	62.66416894	18.40011188
Diopside	0.1001181	0.002866117	0.102984217	102.9842169	2.783064222
Gypsum	0.064682466	0.003267984	0.06795045	67.95045023	4.809362954
Hydroxyapatite	1.89739E-05	0.018070992	0.018089965	18.08996547	99.89511367
Hematite	0.069249782	0.00206493	0.071314712	71.31471223	2.895517907
Kaolinite	0.071491559	0.005637645	0.077129203	77.1292034	7.309351552
Magnetite	0.087120459	0.003514417	0.090634876	90.63487577	3.877555076
Newberyite	0.109277524	0.003267984	0.112545507	112.5455073	2.903699898
Olivine	0.073745617	0.001611778	0.075357395	75.35739521	2.138845607
Orthoclase	0.063897007	0.006769353	0.07066636	70.66636025	9.579314553
Sand	0.087929742	0.001871158	0.089800899	89.80089904	2.083673489
Schriebersite	0.1111905	0.006178802	0.117369302	117.3693023	5.264410941
Siderite	0.052652662	0.003111932	0.055764595	55.76459463	5.58048069
Serpentinite	0.082533096	0.00274475	0.085277847	85.27784664	3.218596937
Struvite	0.116365584	0.006552379	0.122917963	122.917963	5.330692685
Ulexite	0.064555406	0.009305467	0.073860873	73.86087333	12.59864261

Table S4. Mineral Extraction NMR Data. All measurements are from peak integrations in the P31-NMR spectra. The before group represents the solution phase before we extracted the sample with EDTA/NaOH. The after group represents the left-over P in the mineral phase liberated after EDTA/NaOH extraction.

NMR Data				
Before	Phosphite	Phosphate	Pyrophosphate	Triphosphate
Blank	21.6	52.6	23.2	2.6
Calcite	4.5	75	19.7	

Diopside	5.2	64	27.3	2.6	
Gypsum	21.7	63.7	14	0.6	
Hydroxyapatite	3.8	77	18.5	0.6	
Hematite	19.7	49.3	28	3	
Kaolinite	15	53.5	28.3	3.2	
Magnetite	31.6	40.9	25	2.5	
Newberyite	3.1	78.7	18.2		
Olivine	18.1	54.9	24.7	2.3	
Orthoclase	39.7	47.8	11.5	1	
Sand	14.2	54.7	28.4	2.7	
Schriebersite	11.5	52	33.3	3.1	
Siderite	17.6	48.7	30.7	3	
Serpentinite	73.5	18.1	8.2	0.2	
Struvite	7.6	47.4	44.1	0.9	
Ulexite	1.5	73.5	25		
After	Phosphite	Pi	PPi	PPPi	Hypophosphate
Blank	17.1	58.8	24.1		
Calcite	2.6	64.1	33.3		
Diopside		55.5	44.5		
Gypsum	9.1	65	25.9		
Hydroxyapatite		85.5	14.5		
Hematite	37.5	37.8	24.6		
Kaolinite	16.2	54.1	29.7		
Magnetite	35.2	38.8	23.5		
Newberyite	7	70	23		
Olivine	32.8	39.5	27.7		
Orthoclase	14.8	52.9	32.3		
Sand	14.9	51.5	33.5		
Schriebersite	27.2	42.5	15.7	6	8.5
Siderite	23.7	43.1	33.2		
Serpentinite	78	11.4	10.6		
Struvite	8.8	37	51.8	2.2	
Ulexite		57.5	42.5		

References

1. Hazen, R.M., *Paleomineralogy of the Hadean Eon: A preliminary species list.* American Journal of Science, **2013**. 313(9): P. 807-843.
2. Feng, T.; et al., *Evolution of Ephemeral Phosphate Minerals on Planetary Environments.* ACS Earth and Space Chemistry, **2021**. 5(7): P. 1647-1656.
3. Gull, M. and M.A. Pasek, *Catalytic Prebiotic Formation of Glycerol Phosphate Esters and an Estimation of Their Steady State Abundance under Plausible Early Earth Conditions.* Catalysts, **2021**. 11(11).
